# Women’s experiences of care and treatment preferences for perinatal depression: a systematic review

**DOI:** 10.1007/s00737-023-01318-z

**Published:** 2023-05-05

**Authors:** Verity Westgate, Tanya Manchanda, Margaret Maxwell

**Affiliations:** 1 Department of Psychiatry, University of Oxford, Oxford, UK; 2 Nursing, Midwifery and Allied Health Professions Research Unit, University of Stirling, Stirling, UK

**Keywords:** Perinatal, Postnatal, Antenatal, Depression, Treatment

## Abstract

Understanding women’s experiences of care, and treatment preferences, is vital for delivering acceptable and useful services to women with perinatal depression. This systematic review synthesises evidence on care and treatment preferences of women with perinatal depression. This qualitative evidence synthesis uses systematic review methodology. Medline, PsychINFO, CINAHL and EMBASE were searched from January 2011 to October 2021. Search terms fell into five categories: depression, the perinatal period, treatment preferences, experiences of care and qualitative research. Study quality was assessed and thematic analysis was used to synthesise findings. Thirteen papers met the inclusion criteria. Quality of included papers was of moderate to high quality. Five key themes were identified: women prioritise family needs; perinatal-specific care; when care falls short; professional empathy; and tailored care. Clinicians need to enable mothers to prioritise their own well-being. Service providers should ensure that treatment is tailored to the specifics of the perinatal period, providing specialist advice around medication, and therapy that fits with the demands of caring for a new baby.

**Supplementary Information:**

The online version contains supplementary material available at 10.1007/s00737-023-01318-z.

## Background

The perinatal period can be a difficult time for many women. Perinatal depression is the most common perinatal mental health condition and may have requirements for treatment that differ from other perinatal mental health conditions such as perinatal anxiety or postpartum psychosis. Perinatal depression encompasses depression that affects women during pregnancy and in the first year after giving birth (Bailey and Gaskin 2021). Perinatal depression is separate from the “baby blues” which affects up to 70% of mothers but commonly resolves within 2 weeks. Woody et al. (2017) estimated the incidence of perinatal depression to be around 11%, although Slomian et al. (2019) noted that prevalence can depend on how perinatal depression is defined, the country that the woman is in, screening measure thresholds, the tools used for diagnosis and the period of time that is used for diagnosis (e.g. 6 weeks postpartum or up to a year). Symptoms may include low mood, lack of energy and interest in daily activities, sleep difficulties, changes in appetite, poor concentration and feelings of worthlessness, guilt or hopelessness (Dagher et al. 2020). At its worst, perinatal depression can lead to suicide, which is the leading cause of death in women in the year following giving birth, and the fifth most common cause of death during pregnancy and immediately afterwards (MBRRACE-UK 2019).

Perinatal depression may affect outcomes for the child. Dadi et al. (2020) found increased risk of low birth weight and pre-term birth in babies of mothers with depression: adverse birth outcomes such as these are the leading cause for infant and childhood morbidity and mortality (Jacques et al 2019). There may be an association with decreased breastfeeding initiation (Pope and Mazmanian 2016), which may itself have unfavourable impacts on the infant.

Longer-term effects of perinatal depression in offspring have also been identified. Antenatal depression has been shown to correlate with developmental delay in children by the age of 18 months (Deave et al 2008). The observational Avon Longitudinal Study of Parents and Children in the UK found that persistent and severe postnatal depression increases the risk for multiple adverse long-term outcomes for the child (Netsi et al 2018). These outcomes include behavioural problems at age 3.5, lower grades in mathematics at age 16 and higher prevalence of depression at age 18. Tirumalaraju et al. (2020) established that perinatal depression was associated with increased risk of depression in the child at both adolescence and adulthood, and Srinivasan et al. (2020) found an association between mothers who experience perinatal depression and psychotic experiences in their children at age 18.

Perinatal depression is a global problem. The prevalence of common perinatal mental health problems, including perinatal depression, is estimated at around 20% in lower and middle-income counties, and is higher amongst the most marginalised women who lack access to health or social care (McNab et al 2022); this is a higher prevalence than in high-income countries (Atif et al 2015). The World Health Organisation has recently developed a guide for the integration of perinatal mental health into maternal and child services to improve health outcomes in a culturally appropriate way (World Health Organisation 2022). Understanding women’s experiences of care, and treatment preferences, is vital for both commissioners and staff working with women experiencing depression in order to provide an acceptable and useful service.

Four previous systematic reviews have looked at women’s experiences of care and treatment preferences in perinatal mental health more broadly (Dennis and Chung-Lee 2006; Jones et al 2014; Megnin-Viggars et al. 2015; Hadfield and Wittkowski 2017). No recent review has been conducted which specifically focusses on women’s experiences of care, and treatment preferences, for perinatal depression.

The aim of this qualitative evidence synthesis is to answer the following questions:

- What are women’s experiences of care for perinatal depression?
- What are women’s treatment preferences for perinatal depression and why?

## Methods

This review is a qualitative evidence synthesis using thematic analysis. The Enhancing Transparency in Reporting the Synthesis of Qualitative Research: ENTREQ guideline was adhered to (Tong et al. 2012) (see Online Supplemental Material 1). A protocol was not submitted for this review as Master’s level dissertations are not accepted by PROSPERO. The research question was developed using the SPIDER tool (Cooke et al 2012). No ethical approval was required for this project.

### Searching

The following databases were searched from January 2011 to October 2021: Medline (OVID), PsycInfo (OVID), EMBASE (OVID) and CINAHL (EBSCO). Reference lists of eligible studies were hand searched for additional relevant publications as were the previous relevant systematic reviews cited in the background.

A comprehensive search strategy to seek all available studies was developed. Online supplemental material 2 provides the full Medline search strategy.

### Eligibility criteria

This evidence synthesis included qualitative studies reporting primary data, published in English between January 2011 and October 2021. Qualitative methods include but are not limited to: surveys, interviews and focus groups. The richness of the qualitative data presented was considered using the 5-point data richness table developed by Ames et al. (2019). Papers scoring 3 or higher (equating to “reasonable amount of qualitative data that relate to the synthesis objective” (Ames et al 2019, n.p.)) on this scale were included.

Inclusion

- Studies reporting primary qualitative data
- Qualitative studies exploring experiences of care and/or treatment preferences for perinatal depression
- Qualitative studies where the population is women experiencing perinatal depression
- Healthcare contexts directly applicable to the UK (e.g. Western Europe, United States of America, Canada and Australia)
- Papers scoring 3 or higher on Ames et al.’s (2019) data richness table

Exclusion

- Systematic or literature reviews
- Correspondence or short communications
- Studies written in any language other than English
- Studies published before 2011
- Studies looking at a general perinatal population or studies looking at any/all perinatal mental health condition(s) where less than 50% of included participants experience perinatal depression
- Papers scoring 2 or lower on Ames et al.’s (2019) data richness table

### Study selection

Eligible studies were de-duplicated using Endnote reference management software (Endnote Team 2013). The remaining studies were then imported into Rayyan systematic review software (Ouzzani et al 2016) for screening. Two reviewers (VW, TM) independently screened the first 10% of abstracts. The remaining abstracts were screened by one reviewer (VW). The full text of all remaining papers was reviewed against the eligibility criteria by two reviewers (VW, MM), disagreements were resolved by discussion and reasons for exclusion were recorded.

A PRISMA flow diagram (online supplemental file 3) is provided to illustrate the study selection process.

### Data extraction

For each study, the following data was extracted:

- Authors, title, journal
- Study setting: country, date
- Study design: objectives, inclusion and exclusion criteria, sample size and sampling strategy, methodology
- Limitations (identified by author)
- Primary data in the form of quotes from participants

### Analysis

A thematic synthesis was carried out using Thomas and Harden’s framework (2008). It involves three stages: coding the data line by line; categorising the codes into descriptive themes; and categorising these descriptive themes into analytical themes. A coding framework was agreed between VW and MM following independent reading of extracted primary data. The primary data quotes were then subsequently coded and analysed using NVivo software by VW, the codes were grouped into descriptive themes which were then developed into analytical themes.

### Quality assessment and confidence in synthesised findings

The rigour of each study was assessed by one reviewer (VW) using the Critical Appraisal Skills Programme (CASP) qualitative studies checklist. The confidence in the synthesised findings of the papers included in the review was assessed using the Grading of Recommendations Assessment-Development and Evaluation and Confidence in Evidence from Reviews of Qualitative Research (GRADE-CERQual).

### Reflexivity

Reflexivity of researchers is considered key to reporting qualitative research (Noyes et al. 2021). The first author of this review notes her own lived experience of perinatal depression, and that her care and treatment preferences may have influenced the review. However, this also provided a strong motivation for choosing this subject and in wanting to carry it out scrupulously without being led too far by her own thoughts and views. The rigour of the systematic review process itself provides checks and balances, as did the role of the supervisor (MM) as a secondary check on data interpretation.

## Results

The search identified 4035 papers (see online supplemental file 3 for PRISMA diagram). After duplicates were removed, 3085 papers were screened and 62 papers taken forward to full text review. 49 papers were excluded, leaving 13 papers in the final review. Reasons for exclusion at full text review are documented in the PRISMA diagram. A full description of included papers is presented in Table 1.

**Table 1:** Summary of characteristics of included papers

Author	Country	Year	Setting	Antenatal, postnatal or both	Sample size	Methodology	Ethnicity of participants
Byatt et al	USA	2013	Hospital	Both	27	Focus groups	All white
Cook et al	USA	2019	Community	Postnatal	7	Semi-structured interviews	Not given
Feeley et al	Canada	2015	Hospital	Postnatal	30	Semi-structured interview	No ethnicity recorded; 14 Canadian, 5 from other countries
Hadfield et al	UK	2019	Primary care	Postnatal	14	Semi-structured interview	All white British
Iturralde et al	USA	2021	Hospital	Both	30	Telephone focus group	9 white, 10 Asian, 5 Black, 6 Latina
Jarrett	UK	2015	Online	Antenatal	26	Internet discussion forum	No demographic information provided
Millett et al	UK	2018	IAPT	Both	12	Semi-structured interview	5 white British, 3 white other, 1 Black Carribean, 1 Black other, 1 Arab, 1 Asian
Nygaard et al	Denmark	2015	Hospital	Antenatal	8	Semi-structured interview	All Danish, no ethnicity recorded
O’Mahen et al	UK	2014	Online	Postnatal	17	Semi-structured telephone interview	No ethnicity recorded
Rossiter et al	Australia	2012	Community	Postnatal	111	Questionnaire with open-ended questions	No ethnicity recorded
Walton et al	Canada	2014	Hospital	Antenatal	40	Semi-structured interview	31 participants born in Canada, 9 born outside Canada
Young et al	USA	2019	Primary care	Postnatal	20	Semi-structured interview	6 participants were monolingual Spanish speakers, 14 participants spoke English

### Quality assessment results

The quality of the included papers was of moderate to high quality when evaluated by the CASP tool, and no studies were excluded based on this (online supplemental material 5). Two studies were scored poorly for the choice of methodology (Jarrett 2016; Rossiter et al 2012). However, both were included since they included free-text responses and it was felt that they could still contribute to the review as they met the inclusion criteria for Ames et al.’s (2019) data richness table, whilst acknowledging that data from these studies are unlikely to be as rich.

#### Grading of Recommendations Assessment, Development and Evaluation-CERQual

The GRADE-CERQual criteria were used to develop a judgement about confidence in the synthesised findings of the review. All findings were graded as having moderate confidence (online supplemental material 6).

### Theme results

Five main analytic themes were derived from the data: a full list of codes, descriptive themes and analytic themes is presented in online supplemental material 4. Examples of quotes supporting the themes are found in Table 2.

**Table 2:** Quotes supporting themes

Women prioritise family needs	Role of partner	“The father, he’s an important person because he is the one accompanying, he is the one encouraging, he sees the tears, and he does everything … There is nothing for men.” (Feeley et al 2016, p. 124)
	Childcare practicalities and treatment	“If it’s going to cause too much trouble in our lives to get that 7-month-old a babysitter … you have to choose what’s best for your baby. And if that means you miss that [depression treatment] appointment, then that means you miss the appointment.” (Iturralde et al 2021, p. 5)
	Medication and baby	“The main thing really was the health of the baby. I just didn’t want to compromise anything. And then, also, weighing that with my mental health and, you know, what are the risks of going off of this completely? And again, in doing some research, learning that can affect the baby also.” (Walton et al. 2014, p. 496)
Perinatal specific care	Care that validates experiences of being a new mother	“I’ve not been bonding with my baby. So to obviously speak about that and let them know that it is a normal thing” (Hadfield et al 2019, p. 3528)
	Format of therapy needs to meet demands of perinatal period	“It was really helpful but she gave me a lot of homework and realistically I was feeding them around the clock and I just didn’t do it” (Millett et al 2018, p. 9)
	Professionals need training in the specifics of perinatal depression	“I know here they deal more with pregnant women, like they’ve seen like thousands of pregnant women, they give these medication, so that’s why I was like more relaxing.” (Walton et al 2014, p. 498)
	Treatment preferences are affected by the perinatal period	“I wouldn’t be able to forgive myself afterwards if I’d been so selfish and taken the medicine and something would happen to him” (Nygaard et al 2015, p. 488)
	Interventions involving peers are particularly valued	“Being able to attend a support group and meet other moms who are experiencing the same thing, it was really helpful knowing that I wasn’t alone” (Iturralde et al 2021, p. 4)
When care falls short	Focus is outside mother’s perinatal mental health	“[My OBGYN provider] was focused on my medical complications more, but I didn’t feel supported with my depression with her at all” (Iturralde et al 2021, p. 5)
	Professionals sometimes fell short	“I went in needing some help and support but came out being told some people have real problems” (Jarrett 2016, p. 38)
	Options available do not always meet needs	“so pressed for time … never got any counselling or CBT as requested, so had to opt for medication as no other alternative” (Jarrett 2016, p. 38)
	Negative experiences of therapy	“It’s a bit of a production line of, get this person in for their six [sessions]. That’s what it feels like” (Millett et al 2018, p. 11)
	Positive experiences of therapy	“It’s really funny, it’s probably not gonna go away, so I feel the tools she’s given me are really gonna help if I, you know, have moments in my life where that, I feel that way again, I can, I can go back.” (Hadfield et al 2019, p. 3526)
Professional empathy	Importance of supportive professional	"She brightened my days when she visited. I feel that the interaction with a compassionate, qualified and knowledgeable nurse made all the difference. It was such a rewarding and wonderful journey.” (Rossiter et al 2012, p. 93)
	Validation	“It would have been important for somebody to just listen to my concerns … even just reassure me that … it’s okay that that’s happening and it’s not your fault.” (Byatt et al 2013, p. 601)
Tailored care	Medication: the lesser of two evils	“I was in a position where I had to, it was either this [take antidepressants] or don’t go through with it [the pregnancy]” (Walton et al 2014, p. 499)
	Preferences for non drug interventions	“I just hoped, even at that point, I was a great believer in talking therapies because I’d had talking therapies before and other types of therapy, so I sort of knew it was roughly about, it would look at how I was.” (Hadfield et al 2019, p. 3525)
	Preferences for non medicalised support	“alternative or natural remedies seem logical to me” (Battle et al 2013, p. 8)
	Preferences for self management	“I was just looking for ways to manage it really you know instead of digging myself into a hole with it I was looking for ways I could manage it myself … like different techniques I could pick up.” (Hadfield et al 2019, p. 3523)

#### Women prioritise family needs

Women often put the baby’s needs above their own, for example concerns about the effect of taking medication in pregnancy (Nygaard et al 2015). Similar feelings were evident with regards to breastfeeding: medication could be declined due to a belief that breastfeeding was best for the baby (Battle et al 2013). There were concerns about sedating effects of medication interfering with their ability to take care of their baby (Young et al 2019). Alternatively, some mothers recognised that their perinatal depression could have a potentially adverse effect on the baby and that taking medication was the better option (Walton et al. 2014).

Women prioritised looking after their child over attending treatment. Therefore, some women found it helpful to be seen at home (Rossiter et al 2012), others benefitted from the provision of childcare (Hadfield et al 2019). Where childcare was not available, some women felt discouraged from taking their baby with them (Millett et al 2018). Inevitably for some, the lack of childcare meant not receiving treatment (Iturralde et al 2021).

Women noted that their partner could be a key part of their support (Walton et al 2014) but often had concerns that there was little provision to support their partner (Feeley et al 2016) or educate them (Millett et al 2018).

Overall, the women’s focus when making decisions about accepting or attending treatment was on the impact this may have for others, over and above their own needs.

#### Perinatal-specific care

Women reported feeling that their care and treatment were not sufficiently tailored to their specific needs during the perinatal period. For them, validating and understanding their role as a mother was important (Rossiter et al 2012; Byatt et al 2013) and this also helped to normalise their experiences (Hadfield et al 2019). Breastfeeding was seen as a specific stressor and therefore women expressed a specific wish for support in this area (Feeley et al 2016).

Professionals delivering perinatal depression care needed particular perinatal expertise and training in order to help women successfully. Inexperienced professionals were perceived as not meeting women’s needs (Jarrett 2016, p. 38), for example not providing information about the effects of medication and pregnancy (Walton et al 2014, p. 496). Conversely, attending a speciality clinic for advice about medication was found to be valuable (Walton et al 2014).

Women felt that the format of therapy needed to respect the demands on a mother during the perinatal period. For example, therapy that requires an amount of “homework” could be particularly difficult for women to achieve (Iturralde et al. 2021; Millett et al. 2018). The option of online self-help suited some as it could be done at any time and tailored around family needs (Millett et al 2018; O’Mahen et al 2015).

Interventions that involved meeting peers with perinatal depression were valued, particularly for the relationships that were formed (Hadfield et al 2019). These helped to validate experiences, take away loneliness and provided the chance to meet with other women further along in their recovery journey (Cook et al 2019; Iturralde et al 2021, p. 4). An online discussion group intervention was helpful to a number of women (Jarrett 2016, p. 38).

#### When care falls short

Many women’s experiences of care seemed to fall short of what was needed or expected. Obstetric care often lacked attention to mental health (Byatt et al 2013; Iturralde et al 2021; Jarrett 2016; Millett et al 2018). Postnatal care frequently focussed on the baby rather than the mother (Feeley et al 2016). At times an individual professional might let a woman down, only giving a leaflet (Feeley et al 2016; Jarrett 2016)*,* failing to take symptoms seriously (Jarrett 2016) or rushing appointments (Jarrett 2016).

Often the options available to women did not meet their needs. Many complained that there were few alternatives to medication, which was especially frustrating given concerns women had about taking it (Jarrett 2016; Millett et al 2018). Waiting lists for talking therapy exacerbated this (Jarrett 2016).

Some women had negative experiences of therapy. The types of therapy offered did not always suit (Hadfield et al 2019; Millet et al. 2018) and the duration was sometimes insufficient (Millett et al 2018). However, many women had positive experiences of therapy, valuing its input and especially when there was some flexibility around the number of sessions (Hadfield et al. 2019; Millett et al 2018).

#### Professional empathy

Individual professionals could play an important part in providing women with care that met their needs. Validation of a woman’s feelings and experiences around having a new baby was particularly key (Byatt et al 2013). Being supportive and compassionate was an important attribute in healthcare professionals (Iturralde et al 2021; Jarrett 2016; Millett et al 2018; Rossiter et al 2012). One study highlighted the value of “cultural similarity” between the mother and the professional they were seeing, if the mother was from a minority background (Iturralde et al 2021, p. 5).

#### Tailored care

Patients expressed different preferences for the use of medication, talking therapies, and non-medical and social support options. Ultimately it was clear that for many women, feeling like someone might be there to take care of *them* was important.

Views about medication varied considerably. Many women preferred to avoid medication in pregnancy or whilst breastfeeding (Battle et al 2013; Iturralde et al 2021). Some women were more conflicted but recognised that it could be helpful (Feeley et al 2016, p. 53). Others had a clear preference for medication (Iturralde et al 2021) or recognised that they might choose medication if their symptoms became severe (Walton et al 2014, p. 499).

Some women expressed a preference for counselling or therapy (Battle et al. 2013; Byatt et al. 2013; Feeley et al 2016; Hadfield et al 2019). Preferences for individual or group support varied (Feeley et al 2016). One woman suggested that check-ins would be helpful, to make her feel cared for (Byatt et al 2013, p. 602).

Many preferences were expressed for support beyond the medical profession. Support from friends or family was favoured and often helped women to feel looked after (Feeley et al 2016). Others wanted alternative remedies (Battle et al 2013, p. 8), or help with breastfeeding (Feeley et al 2016, p. 125). Several women preferred to use their own resources to deal with perinatal depression, such as self-management (Hadfield et al 2019), exercise (Battle et al 2013, p. 8) or creative activities (Feeley et al 2016, p. 125).

## Discussion

This review included 13 papers of moderate to high quality. A number of key themes were identified regarding the experiences of care, and treatment preferences, for women with perinatal depression. There was a commonality to the themes despite considerable heterogeneity in the study populations, study settings and methodologies, which suggests transferability between settings of care for perinatal depression more generally. There was little overlap of papers with the previous systematic reviews, due to later time-period and the specific focus on perinatal depression. Ethnic diversity amongst participants was generally poor.

Mothers experiencing perinatal depression put their baby first when making decisions about their health needs and treatment. This is reflected in rationales given for either taking or not taking medications (Battle et al 2013; Feeley et al 2016; Iturralde et al 2021; Walton et al 2014), and also when it comes to engaging in therapy if the setup does not meet their needs as caregivers to their baby (Iturralde et al 2021; Millett et al 2018; Rossiter et al 2012).

At the same time, mothers with perinatal depression want someone to acknowledge their needs and take care of them (Byatt et al 2013). This might be a member of clinical staff who is able to take account of and respond to their individual preferences for treatment (Iturralde et al 2021; Jarrett 2016; Millett et al 2018; Rossiter et al 2012), or it might be outside the clinical professions (Battle et al 2013; Feeley et al 2016). Sometimes this need for women to feel cared for and supported was missed by healthcare professionals (Byatt et al. 2013; Feeley et al 2016; Jarrett 2016), and this is an area where clinicians might be more proactive in order to improve women’s experiences of care. This corroborates Megnin-Viggars et al.’s (2015) conclusion that women sought but did not often receive individualised treatment in line with their preferences. Similarly, Dennis and Chung-Lee (2006, p. 329) concluded that each mother will have different requirements for care, suggesting that there has been little change in the priorities for women with perinatal depression.

It is clear from the studies included in this review that individualised care should be informed by the specifics of the perinatal period. There is a need to make treatment accessible for women who are caregivers first and foremost and who may consequently struggle to attend appointments or carry out therapy homework (Hadfield et al 2019; Iturralde et al 2021; Millett et al 2018; Rossiter et al 2012). This is upheld by Lever Taylor et al.’s (2021) investigation of models of community care for women with perinatal mental health problems, women clearly valued the expertise and differing attitudes of staff in specific perinatal services as opposed to general community mental health teams.

Whilst a previous review (Megnin-Viggars et al. 2015) suggested that concerns about medication could indicate an unmet need with regards to the provision and communication of information around risks and benefits in pregnancy and whilst breastfeeding, this review found a frequent preference for non-pharmacological treatment (Battle et al 2013; Byatt et al 2013; Feeley et al 2016; Hadfield et al 2019). This should be a clear priority for those working with women with perinatal depression.

Validation of the mother’s feelings and experiences is vital, whether by a professional or in a support group, but it may be easier for this to occur amongst a peer group (Byatt et al 2013; Cook et al 2019; Hadfield et al 2019; Iturralde et al 2021; Jarrett 2016). Validation by a therapist from a similar ethnic background is highly valued (Iturralde et al 2021). Jones et al (2014)’s meta-ethnography of the impact of peer support in perinatal mental illness found that peer support helped to overcome isolation, provided validation and helped to give women a way forward, but that the focus needs to be specific to meet their needs.

### Limitations of the review

This review has some limitations. Grey literature and unpublished material was not searched which means some relevant material may have been missed. In addition, it was not possible to publish a review protocol, as PROSPERO do not register master’s dissertations.

Methodological rigour was assessed using the CASP guidelines and confidence in the synthesised findings was evaluated using GRADE-CERQual, but the results may be limited by data quality within the studies. Broadly, studies were methodologically robust but it was common not to explore the relationship between researcher and participants, or this was not explicitly documented. Publication bias may also have affected the results that were included.

This review was limited to healthcare contexts directly applicable to the UK which may limit its utility beyond similar Westernised countries, although one study did look explicitly at barriers to engaging with treatment for perinatal depression across ethnic groups (Iturralde et al 2021). Even within these contexts, participant diversity was limited and not reflective of westernised countries.

### Future research

Studies in this synthesis came from a variety of settings, often centred on a specific intervention but no studies had been conducted in a specialist community perinatal team. As these teams become more established, it would be valuable to see if themes identified in this study are replicated in this environment. In addition, no studies were based in a Mother and Baby Unit (MBU) and preferences for or against inpatient treatment were not mentioned; this mode of treatment is a rare but important one for women with the most severe perinatal depression. Both specialist community perinatal teams and MBUs often offer formalised peer support through employed peer support workers, and research could look at their role in women’s experiences of care and treatment preferences.

Studies included in this review reflect the historic focus of studies of depression in the perinatal period on postnatal depression. Since specialist perinatal teams concentrate on the period of pregnancy to 12 months post-partum, it would be constructive to consider both antenatal and postnatal periods, following women longitudinally to assess whether experiences of care and treatment preferences change over time or differ between the antenatal and postnatal periods.

Ethnic diversity was limited in the studies included with the exception of one study looking at this. Future research should attempt to ensure a better diversity of participants as this may have implications for drawing conclusions about women’s experiences of care and treatment preferences.

### Conclusion and implications for policy and practise

This evidence synthesis has examined experiences of care, and treatment preferences, for women with perinatal depression and updates previous reviews looking at these themes.

Mothers experiencing perinatal depression put their baby first when making decisions about their health needs and treatment, but also want someone to acknowledge their needs and take care of them. Sometimes this need for care and support is missed by healthcare professionals. Women want healthcare professionals to take account of and respond to their individual preferences for treatment, and especially for treatment opportunities and modalities to fit with or be flexible enough for mothers caring for a new baby. Women value input from specialists in perinatal mental health and also prefer non-pharmacological approaches as interventions, including peer support.

This review has considerable utility for clinicians and service providers working with women with perinatal depression. Service providers and clinicians should be aware of the individual nature of treatment preferences for perinatal depression and ensure that a range of evidence-based treatments are offered. They should ensure that treatment is tailored to the specifics of the perinatal period, providing specialist advice around medication, and therapy that fits with the lifestyle and demands of caring for a new baby. All clinicians should be aware of the importance of their role as individuals, which can make or break how a woman perceives her experiences of care. The findings of this review have the potential to inform the implementation of the priorities for perinatal mental health set by NHS England in the NHS Mental Health Implementation Plan for 2019/20–2023/24 (2019) and similar plans in other countries.

## Supplementary Information

Below is the link to the electronic supplementary material.

Supplementary file1 (PDF 620 KB)

Supplementary file2 (PDF 425 KB)

Supplementary file3 (PDF 704 KB)

Supplementary file4 (PDF 610 KB)

Supplementary file5 (PDF 594 KB)

Supplementary file6 (PDF 620 KB)
