# Supplementary material for: Women’s experiences of care and treatment preferences for perinatal depression: a systematic review
Source: Arch Womens Ment Health. 2023 May 5;26(3):311–9. doi: 10.1007/s00737-023-01318-z (PMC10191949; doi:10.1007/s00737-023-01318-z)
Supplement: Supplementary file 2 — Supplementary file2 (PDF 425 KB) [file 737_2023_1318_MOESM2_ESM.pdf]

Online Supplemental Material 2

Medline search strategy

1. (pregnan* or perinatal or antenatal or postnatal or maternal or trimester or antepartum or prepartum or postpartum).mp. [mp=title, abstract, original title, name of substance word, subject heading word, floating sub-heading word, keyword heading word, organism supplementary concept word, protocol supplementary concept word, rare disease supplementary concept word, unique identifier, synonyms]
2. exp Pregnancy/
3. exp Postpartum Period/
4. 1 or 2 or 3
5. (depress* or postpartum depression or postnatal depression or antenatal depression).ab,ti.
6. exp Depression, Postpartum/
7. exp Depression/
8. 5 or 6 or 7
9. (Treatment preference or Patient preference or Patient choice or Treatment option or Patient satisfaction or Patient attitude or Treatment refusal or Treatment planning or Compliance).ab,ti.
10. exp Patient Preference/
11. exp Patient Satisfaction/
12. exp Patient Compliance/
13. exp Treatment Refusal/
14. 9 or 10 or 11 or 12 or 13
15. (Experiences of care or Quality of care or Health care access or Health care quality or Patient centred care).ab,ti.
16. exp "Quality of Health Care"/
17. exp Health Services Accessibility/
18. exp Patient-Centered Care/
19. exp Patient Care/
20. 15 or 16 or 17 or 18 or 19
21. 14 or 20
22. (qualitative or interview* or semi structured interview or structured interview or unstructured interview or telephone interview or discussion or focus group* or ethno* or field work or fieldwork or field stud*).ab,ti.
23. exp Qualitative Research/
24. exp Interview/
25. exp Focus Groups/
26. 22 or 23 or 24 or 25
27. 4 and 8 and 21 and 26
28. limit 27 to yr="2011 - 2021"

Westgate et al (2022), Women's experiences of care and treatment preferences for perinatal depression: a systematic review, Archives of Women's Mental Health. Corresponding author: Verity Westgate, Department of Psychiatry, University of Oxford, verity.westgate@psych.ox.ac.uk
