# Supplementary material for: Women’s experiences of care and treatment preferences for perinatal depression: a systematic review
Source: Arch Womens Ment Health. 2023 May 5;26(3):311–9. doi: 10.1007/s00737-023-01318-z (PMC10191949; doi:10.1007/s00737-023-01318-z)
Supplement: Supplementary file 3 — Supplementary file3 (PDF 704 KB) [file 737_2023_1318_MOESM3_ESM.pdf]

Online supplemental material 3:

PRISMA 2020 flow diagram for new systematic reviews which included searches of databases and registers only

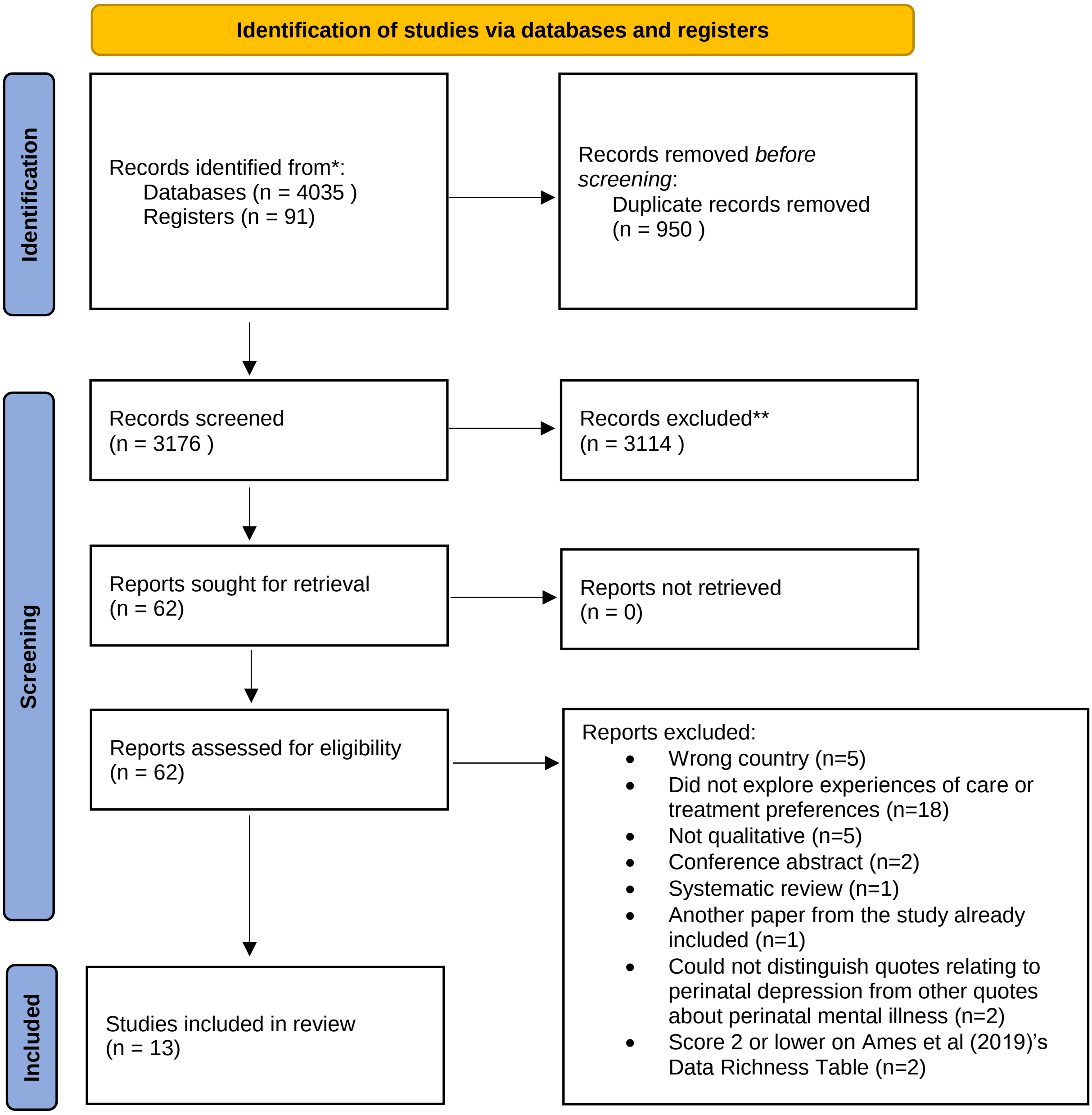

From: Page MJ, McKenzie JE, Bossuyt PM, Boutron I, Hoffmann TC, Mulrow CD, et al. The PRISMA 2020 statement: an updated guideline for reporting systematic reviews. BMJ 2021;372:n71. doi: 10.1136/bmj.n71

For more information, visit: http://www.prisma-statement.org/

Westgate et al (2022), Women's experiences of care and treatment preferences for perinatal depression: a systematic review, Archives of Women's Mental Health. Corresponding author: Verity Westgate, Department of Psychiatry, University of Oxford, verity.westgate@psych.ox.ac.uk
