# Supplementary material for: Women’s experiences of care and treatment preferences for perinatal depression: a systematic review
Source: Arch Womens Ment Health. 2023 May 5;26(3):311–9. doi: 10.1007/s00737-023-01318-z (PMC10191949; doi:10.1007/s00737-023-01318-z)
Supplement: Supplementary file 4 — Supplementary file4 (PDF 610 KB) [file 737_2023_1318_MOESM4_ESM.pdf]

Online Supplemental Material 4

Thematic structure

Analytic themes	Descriptive themes	Codes
Women prioritise family needs	Role of partner	Desire for partner to be involved in decision-making
		Desire for education of partner
	Childcare practicalities and treatment	Location of treatment
		Lack of childcare means cannot participate in therapy
		Provision of childcare helpful
		Difficult to find childcare
		Able to take child but not well set up for this
	Medication and baby	Concerns about sedating effects of medication whilst caring for baby
		Concerns about medication in pregnancy
		Concerns about medication and breastfeeding
Perinatal-specific care	Care that validates experiences of being a new mother	Value of work focussing on parenting
		Value of work focussing on bond with baby
		Validation of perinatal experiences is helpful
		Would like breastfeeding coach
	Format of therapy needs to meet demands of perinatal period	Online therapy can be done at any time
		Lack of time to engage with new resources
		Therapy homework is a burden
	Professionals need training in the specifics of perinatal depression	Inexperienced professionals
		Lacked information about the effects of medication and pregnancy
		Value of being able to attend a speciality clinic for advice about medication
	Treatment preferences are affected by the perinatal period	Concerns about medication in pregnancy
		Concerns about medication and breastfeeding
	Interventions involving peers are particularly valued	Value of peer relationships built in group therapy
		Discussion board helpful
When care falls short	Focus is outside mother's perinatal mental health	Obstetric team focus on pre-pregnancy and physical health

Westgate et al (2022), Women's experiences of care and treatment preferences for perinatal depression: a systematic review, Archives of Women's Mental Health. Corresponding author: Verity Westgate, Department of Psychiatry, University of Oxford, verity.westgate@psych.ox.ac.uk

		Postnatal care focusses on baby not mother
	Professionals sometimes fell short	Only given a leaflet
		Symptoms not taken seriously/not understood
		Sympathy and understanding but nothing more
		Pointless form-filling
		Inexperienced professionals
		Appointments were rushed
		Professionals did not ask about mental health needs
	Options available do not always meet needs	Long waiting list so no alternative to medication
		Few alternatives to medication
		CBT superficial
		Telephone assessment difficult as hard to relate to professional
	Negative experiences of therapy	Therapy homework was a burden around the needs of the baby
		Motivation made participating in therapy difficult
		Lack of flexibility with number of sessions
		Group therapy like being at school
		CBT superficial
	Positive experiences of therapy	Therapy gives the tools
		Positive change as a result of therapy
		Flexibility around the sessions
		Online therapy could be done at any time
Professional empathy	Importance of supportive professional	Importance of supportive professional
		Value of therapist that speaks same language
		Value of cultural similarity with therapist
	Validation	Importance of being heard
Tailored care	Medication: the lesser of two evils	Preference to avoid medication in pregnancy
		Preference for medication
		Preference for medication if symptoms become severe
		Conflicting feelings about medication
	Preferences for non-drug interventions	Preference for support group
		Preference for individual rather than group support

Westgate et al (2022), Women's experiences of care and treatment preferences for perinatal depression: a systematic review, Archives of Women's Mental Health. Corresponding author: Verity Westgate, Department of Psychiatry, University of Oxford, verity.westgate@psych.ox.ac.uk

		Preference for counselling/therapy over medication
		Preference for information in the antenatal period
		Preference for check-ins
	Preferences for non-medicalised support<br><br>Preferences for self-management	Preference for support from friends or family
		Preference for alternative remedies
		Preference for self-management
		Preference for creative activities
		Preference for exercise

Westgate et al (2022), Women's experiences of care and treatment preferences for perinatal depression: a systematic review, Archives of Women's Mental Health. Corresponding author: Verity Westgate, Department of Psychiatry, University of Oxford, verity.westgate@psych.ox.ac.uk
