# Supplementary material for: Women’s experiences of care and treatment preferences for perinatal depression: a systematic review
Source: Arch Womens Ment Health. 2023 May 5;26(3):311–9. doi: 10.1007/s00737-023-01318-z (PMC10191949; doi:10.1007/s00737-023-01318-z)
Supplement: Supplementary file 5 — Supplementary file5 (PDF 594 KB) [file 737_2023_1318_MOESM5_ESM.pdf]

## Online supplemental material 5

## Rigour/Risk of Bias Assessment of Included Papers

| **Authors** | Was there a clear statement of the aims of the research? | Is a qualitative methodology appropriate? | Was the research design appropriate to the aims of the research? | Was the recruitment strategy appropriate to the aims of the research? | Was the data collected in a way that addressed the research issue? | Has the relationship between researcher and participants been adequately considered? | Have ethical issues been taken into consideration? | Was the data analysis sufficiently rigorous? | Is there a clear statement of findings? | How valuable is the research? |
|---|---|---|---|---|---|---|---|---|---|---|
| Battle et al | Y | Y | Y | CT | Y | N | CT | Y | Y | Y |
| Byatt et al | Y | Y | Y | CT | Y | N | CT | Y | Y | Y |
| Cook et al | Y | Y | Y | CT | Y | N | CT | Y | Y | Y |
| Feeley et al | Y | Y | Y | Y | Y | N | Y | Y | Y | Y |
| Hadfield et al | Y | Y | Y | Y | Y | Y | Y | Y | Y | Y |
| Iturralde et al | Y | Y | Y | CT | Y | N | Y | Y | Y | Y |
| Jarrett | Y | Y | N | Y | Y | N | Y | Y | Y | Y |
| Millett et al | Y | Y | Y | Y | Y | Y | Y | Y | Y | Y |
| Nygaard et al | Y | Y | Y | Y | Y | N | Y | Y | Y | Y |

| | | | | | | | | | | |
|---|---|---|---|---|---|---|---|---|---|---|
| O'Mahen et al | Y | Y | Y | Y | Y | N | Y | Y | Y | Y |
| Rossiter et al | Y | Y | N | Y | Y | N | Y | Y | Y | Y |
| Walton et al | Y | Y | Y | Y | Y | N | Y | Y | Y | Y |
| Young et al | Y | Y | Y | Y | Y | N | CT | Y | Y | Y |

Key: Y=Yes, N=No, CT=Can't tell.
