# Supplementary material for: Women’s experiences of care and treatment preferences for perinatal depression: a systematic review
Source: Arch Womens Ment Health. 2023 May 5;26(3):311–9. doi: 10.1007/s00737-023-01318-z (PMC10191949; doi:10.1007/s00737-023-01318-z)
Supplement: Supplementary file 6 — Supplementary file6 (PDF 620 KB) [file 737_2023_1318_MOESM6_ESM.pdf]

Online Supplemental Material 6

Confidence in Synthesised Findings Using the GRADE-CERQual Framework

Summary of review finding	Papers contributing to finding	Methodological limitations	Coherence	Adequacy	Relevance	CER-Qual confidence assessment	Explanation of the CER-Qual evidence
Treatment needs to take account of whole family. This includes thinking about the role of the partner, making sure that the practicalities of treatment are accessible for women caring for babies and keeping the baby in mind when making decisions about medication.	Battle et al 2013; Feeley et al 2016; Hadfield et al 2019; Iturralde et al 2021; Millett et al 2018; Nygaard et al 2015, Rossiter et al 2012; Walton et al 2014; Young et al 2019.	Moderate concerns	No concerns	Minor concerns	No concerns	Moderate confidence	There are moderate concerns regarding methodological limitations. Nine studies contributed to this review finding. Two studies were unclear around their recruitment strategy. Seven studies failed to consider reflexivity between researcher and participants. One study only used a survey with free-text responses, limiting data richness.
Treatment needs to be tailored to the specifics of the	Byatt et al 2013; Cook et al 2019; Feeley et al 2016;	Moderate concerns	No concerns	Minor concerns	No concerns	Moderate confidence	There are moderate concerns regarding methodological limitations. Eleven studies contributed to this review

perinatal period. Professionals need specific perinatal experience, as decisions around treatment can be affected by the perinatal period. Women find that treatment that focuses on the role of being a mother or in peer support interventions can be helpful.	Hadfield et al 2019; Iturralde et al 2021; Jarrett 2016; O'Mahen et al 2015; Millett et al 2018; Nygaard et al 2015; Rossiter et al 2012; Walton et al 2014.						finding. Three studies were unclear around their recruitment strategy. Nine studies failed to consider reflexivity between researcher and participants. One study only used a survey with free-text responses and one study used responses on an internet discussion forum, limiting data richness.
Experiences of care sometimes fall short. Professionals themselves can fall short and the focus can sometimes be outside the mother's perinatal mental health. The options available do not always	Byatt et al 2013; Feeley et al 2016; Hadfield et al 2019; Iturralde et al 2021; Jarrett 2016; Millett et al 2018.	Moderate concerns	No concerns	Minor concerns	No concerns	Moderate confidence	There are moderate concerns regarding methodological limitations. Six studies contributed to this review finding. One study was unclear around their recruitment strategy. Four studies failed to consider reflexivity between researcher and participants. One study only used responses on an internet discussion forum, limiting data richness.

meet the needs of perinatal women. Women have both positive and negative experiences of therapy.							
Important role of individual professional in providing care that meets needs. Being supportive and validating women's experiences is key and there is value in cultural similarity between therapist and patient.	Byatt et al 2013; Iturralde et al 2021; Jarrett 2016; Millett et al 2018; Rossiter et al 2012.	Moderate concerns	No concerns	No concerns	No concerns	Moderate confidence	There are moderate concerns regarding methodological limitations. Five studies contributed to this review finding. Two studies were unclear around their recruitment strategy. Four studies failed to consider reflexivity between researcher and participants. One study only used a survey with free-text responses and one study used responses on an internet discussion forum, limiting data richness.
Preferences for the type of support and treatment are individual. Patients express different preferences around	Battle et al 2013; Byatt et al 2013; Feeley et al 2016; Hadfield et al 2019; Walton et al 2014.	Moderate concerns	No concerns	No concerns	No concerns	Moderate confidence	There are moderate concerns regarding methodological limitations. Five studies contributed to this review finding. Four studies were unclear around their recruitment strategy. Four studies failed to consider reflexivity between researcher and participants.

medication, the type of therapy and around finding different avenues of support outside the medical profession.							
